# Adaptive Changes in the Sensitivity of the Dorsal Raphe and Hypothalamic Paraventricular Nuclei to Acute Exercise, and Hippocampal Neurogenesis May Contribute to the Antidepressant Effect of Regular Treadmill Running in Rats

**DOI:** 10.3389/fnbeh.2017.00235

**Published:** 2017-11-24

**Authors:** Ayu Nishii, Seiichiro Amemiya, Natsuko Kubota, Takeshi Nishijima, Ichiro Kita

**Affiliations:** Laboratory of Behavioral Neuroscience, Department of Human Health Science, Tokyo Metropolitan University, Hachioji, Japan

**Keywords:** DRN, PVN, neurogenesis, depression, immunohistochemistry

## Abstract

Increasing clinical evidence suggests that regular physical exercise can prevent or reduce the incidence of stress-related psychiatric disorders including depressive symptoms. Antidepressant effect of regular exercise may be implicated in monoaminergic transmission including serotonergic transmission, activation of the hypothalamic-pituitary-adrenal (HPA) axis, and hippocampal neurogenesis, but few general concepts regarding the optimal exercise regimen for stimulating neural mechanisms involved in antidepressant properties have been developed. Here, we examined how 4 weeks of treadmill running at different intensities (0, 15, 25 m/min, 60 min/day, 5 times/week) alters neuronal activity in the dorsal raphe nucleus (DRN), which is the major source of serotonin (5-HT) neurons in the central nervous system, and the hypothalamic paraventricular nucleus (PVN), in which corticotropin-releasing factor (CRF) neurons initiate the activation of the HPA axis, during one session of acute treadmill running at different speeds (0, 15, 25 m/min, 30 min) in male Wistar rats, using c-Fos immunohistochemistry. We also examined neurogenesis in the hippocampus using immunohistochemistry for doublecortin (DCX) and assessed depressive-like behavior using the forced swim test after regular exercise for 4 weeks. In the pre-training period, acute treadmill running at low speed, but not at high speed, increased c-Fos positive nuclei in the DRN compared with the sedentary control. The number of c-Fos positive nuclei in the PVN during acute treadmill running was increased in a running speed-dependent manner. Regular exercise for 4 weeks, regardless of the training intensity, induced an enhancement of c-Fos expression in the DRN during not only low-speed but also high-speed acute running, and generally reduced c-Fos expression in the PVN during acute running compared with pre-training. Furthermore, regular treadmill running for 4 weeks enhanced DCX immunoreactivity in the hippocampal dentate gyrus (DG), and resulted in decreased depressive-like behavior, regardless of the training intensity. These results suggest that long-term repeated exercise, regardless of the training intensity, improves depressive-like behavior through adaptive changes in the sensitivity of DRN and PVN neurons to acute exercise, and hippocampal neurogenesis.

## Introduction

There is increasing clinical evidence that physical activity and exercise can reduce or prevent the incidence and symptoms of stress-related psychiatric disorders including depression (Paluska and Schwenk, 2000; Daley, 2008; Duman et al., 2008; Ströhle, 2009; Matta Mello Portugal et al., 2013; Stanton and Reaburn, 2014). Several studies have suggested that depression is similarly affected by selective serotonin reuptake inhibitors (SSRIs) and physical exercise (Babyak et al., 2000; Russo-Neustadt et al., 2001; Carek et al., 2011). Increased serotonin (5-HT) synthesis, metabolism, and release have been observed during or following exercise (Chaouloff, 1997; Gomez-Merino et al., 2001; Meeusen et al., 2001).

Several studies have indicated that neuronal activation in the dorsal raphe nucleus (DRN), which is in the midbrain and is one of the major sources of (5-HT) neurons in the central nervous system, is implicated in antidepressant properties (Owens and Nemeroff, 1994; Artigas et al., 1996; Berton and Nestler, 2006; Pittenger and Duman, 2008; Albert et al., 2014; Mahar et al., 2014). The central serotonergic system originated from the DRN has widespread projections to emotion-related brain regions including the amygdala, hippocampus, basal ganglia and cortex (Vertes, 1991; Jacobs and Azmitia, 1992), and is associated with antidepressant properties.

Symptoms of depression associated with dysregulation of the hypothalamic-pituitary-adrenal (HPA) axis, which is initiated by activation of corticotropin-releasing factor (CRF) neurons in the hypothalamic paraventricular nucleus (PVN; Lee et al., 1987; Dunn and Berridge, 1990; Bremner et al., 1997; Arborelius et al., 1999; Baker et al., 1999; Bakshi and Kalin, 2000; Brouwer et al., 2005). CRF neurons in the PVN project to extrahypothalamic brain regions such as the DRN, locus coeruleus, amygdala, and bed nucleus of the stria terminalis, which are regions involved in mood, anxiety, and stress responses (Gray, 1993; Lee and Davis, 1997; Anderson and Shivakumar, 2013). It has been reported that physical exercise activates the HPA axis, depending on the type, intensity, and duration of exercise (Vale et al., 1981; Chennaoui et al., 2002; Kawashima et al., 2004; Yanagita et al., 2007; Otsuka et al., 2016). Thus, regular physical exercise may mediate depressive behavior through adaptive changes (i.e., sensitivity) in the neuronal activity in the DRN and PVN.

Furthermore, an increase in hippocampal neurogenesis may be required for the antidepressant action mediated by regular physical exercise (Dranovsky and Hen, 2006; Ernst et al., 2006; Becker and Wojtowicz, 2007; Zainuddin and Thuret, 2012; Yuan et al., 2015), and hippocampal neurogenesis could be enhanced by serotonergic neurotransmission and by inhibition of the HPA axis (Jacobs, 2002; Santarelli et al., 2003; Balu and Lucki, 2009). Therefore, regular exercise may produce its antidepressant effect through hippocampal neurogenesis, as well as changes in the sensitivity of neuronal activity in the DRN and PVN.

Although accumulating evidence suggests that regular exercise has beneficial effects on mood, stress responses, and stress-related psychiatric disorders including depression (Paluska and Schwenk, 2000; Duman et al., 2008; Ströhle, 2009; Greenwood and Fleshner, 2011), few general concepts regarding the optimal exercise regimen for stimulating neural mechanisms involved in antidepressant properties have been developed (Leasure and Jones, 2008; Ströhle, 2009). Indeed, the exercise regimens in previous studies have varied widely, and clinical studies have shown that the beneficial effects of physical exercise depend on the parameters of the exercise, including the intensity, duration, and type of exercise (Daley and Welch, 2004; Leasure and Jones, 2008; Rendi et al., 2008; Bibeau et al., 2010; Matta Mello Portugal et al., 2013; Stanton and Reaburn, 2014). In the present study, we examined the effects of 4 weeks of regular treadmill running at different intensities on neuronal activity in the DRN and PVN during a bout of acute treadmill running in rats using immunohistochemistry for c-Fos and on hippocampal neurogenesis using immunohistochemistry for doublecortin (DCX). We decided to test an acute marker, c-Fos, to investigate whether the adaptive changes in the sensitivity of the neuronal activity to acute exercise were induced by regular physical exercise. We also performed the forced swim test to assess depressive-like behavior after sessions of regular treadmill running. Our results suggest that adaptive changes in the neuronal activity in the DRN and PVN and hippocampal neurogenesis could contribute to the antidepressant effects of regular physical exercise.

## Materials and Methods

### Animals

Sixty adult male Wistar rats (weighing 240–280 g at the beginning of the experiment) were used for the experiments. All rats were individually caged and maintained under controlled colony conditions of temperature (23°C) and light (12-h light/12-h dark cycle, lights on at 05:00 h). Rats were given *ad libitum* access to food and water. All experimental procedures were approved by the Animal Experimentation Ethics Committee of Tokyo Metropolitan University. All efforts were made to minimize animal suffering and the number of animals used.

### Experimental Procedures

**Figure 1:**
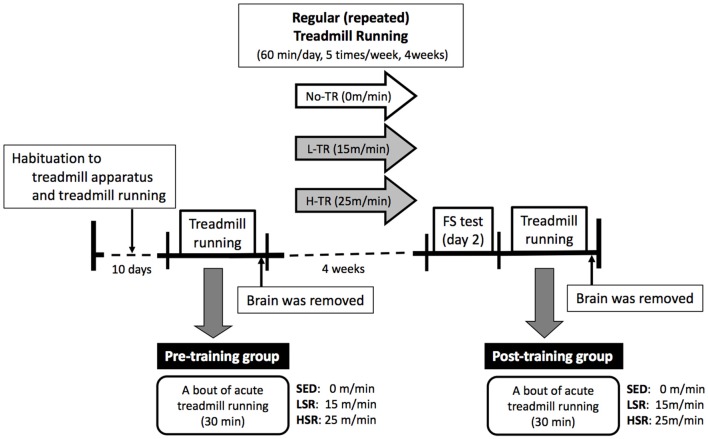
Time line of the experiment. The rats were habituated to the treadmill apparatus and treadmill running for 10 days. Then, a session of 30-min treadmill running (LSR, low-speed runners; HSR, high-speed runners) or sitting (SED, sedentary controls) on the treadmill was performed in half of the rats as the pre-training group. The brains of rats were removed following this single session of treadmill running. The remaining half of the rats performed regular treadmill running at different intensities for 4 weeks (H-TR, high intensity; L-TR, low intensity; No-TR, sedentary; 60 min/day, 5 times/week). After the training session, the rats performed a behavioral test (i.e., forced swim test) and acute treadmill running (the same regimen as in the pre-training group) as the post-training group. Two or more days after the behavioral testing, the brains were removed following a session of acute treadmill running.

A time line of the experiment is presented in Figure 1. All of treadmill procedures were conducted in the dark period of the light/dark cycle. All rats were habituated to the treadmill apparatus and treadmill running for 10 days according to previous studies (Ohiwa et al., 2006; Soya et al., 2007; Otsuka et al., 2016). The 10-day running habituation protocol comprised running on a motor driven treadmill at a 0° incline with an incremental increase in speed (from 10 m/min to 25 m/min) and duration (from 15 min to 60 min). If not keeping up the pace with the treadmill rate, the rats received a mild but aversive foot shock provided by shock grids at the rear of the treadmill to maintain rats’ running at the correct exercise intensity. Very few foot shocks were administrated during each habituation session. Some rats were excluded when, in rare cases, they received excessive numbers of foot shocks, or refused to run. After the habituation period, half of the rats performed a bout of 30-min session of treadmill running (as the pre-training group). The rats were randomly assigned to one of three groups for a single session of acute treadmill running: sedentary controls (SED, *n* = 10), low-speed runners (LSR; 15 m/min, *n* = 10), and high-speed runners (HSR; 25 m/min, *n* = 10). Rats in the last two groups of runners (LSR and HSR) performed 30 min of treadmill running at the assigned speed without foot shocks. The SED group was placed on a stationary treadmill for 30 min. As described below in “Immunohistochemistry” sections, rats were anesthetized and sacrificed following the acute treadmill running, and brains were removed for immunohistochemistry. The lactate threshold (LT) in rats is at a running speed of approximately 20 m/min (Saito and Soya, 2004; Soya et al., 2007), and thus, low- and high-speed running corresponded to sub- and supra-LT, respectively. The other half of the group of rats performed 4 weeks of regular treadmill running (as the post-training group). The rats in the post-training group were also randomly assigned to one of three groups at different intensities (high intensity, H-TR, 25 m/min, *n* = 10; low intensity, L-TR, 15 m/min, *n* = 10; No-TR, sedentary, just sitting on the treadmill, *n* = 10; 60 min/day, 5 times/week) for the treadmill training. After the training session, the rats performed a behavioral test and acute treadmill running (as the post-training group). Behavioral testing (i.e., forced swim test) was performed at least 2 days after the last training to evaluate depressive-like behavior. Two days or more after the behavioral testing, each rat performed a session of 30-min treadmill running without foot shocks at the same intensity as the training session to determine the sensitivity of neuronal activity to the acute treadmill running. This regimen was the same as the acute treadmill running in the pre-training group. Rats were sacrificed following the 30-min treadmill running or sitting on the treadmill, and brains were removed for immunohistochemistry.

### Behavioral Tests

The forced swim test was administered as previously described (Duman et al., 2008; Snyder et al., 2011; Otsuka et al., 2016). The forced swim procedure consists of an initial 15-min pre-test swim session on day 1 and a 6-min swim test 24 h after from the pre-test session. Each rat was individually placed in a Plexiglas cylinder (25 cm diameter, 50 cm height) containing water (35 cm deep, 23–25°C) and monitored via a video camera for 6 min on day 2. The immobility time during the last 5 min of a 6-min swim test was scored by an observer as a measure of depressive-like behavior. The observer was blinded to the condition of regular exercise. Immobility is defined as the absence of movement but includes the presence of movements that are required to keep the head above water.

### Immunohistochemistry

Ninety minutes after the start of the 30-min acute treadmill running, all rats in both pre- and post-training groups were deeply anesthetized with intraperitoneal injection of sodium pentobarbital (50 mg/kg) and perfused transcardially with heparin solution (1000 U/l, 0.9% saline), followed by ice-cooled 4% paraformaldehyde, 0.1% glutaraldehyde and 0.2% picric acid in 0.1 M phosphate-buffered saline (PBS, pH 7.4). The rat brains were removed and post-fixed in the same fixative solution without glutaraldehyde for 24 h at 4°C, and then immersed in a phosphate-buffered 30% sucrose solution with 0.1% sodium azide for 24–48 h for cryoprotection. The brains were then frozen and cut in the coronal plane (six series of 40-μm thick sections) on a microtome and collected in 0.1 M PBS with 0.1% sodium azide.

One of six series of sections was selected for immunohistochemistry for c-Fos, which is a well-known transcription factor used for functional marker of neuronal activity (Curran and Franza, 1988; Dragunow and Faull, 1989; Sheng and Greenberg, 1990; Kovács, 1998). In the post-training group, an adjacent series was also used for staining for DCX, a marker of immature neurons that has been validated for assessing neurogenesis (Rao and Shetty, 2004; Couillard-Despres et al., 2005; Nishijima et al., 2013). Immunohistochemical visualization of c-Fos in the DRN and PVN was performed by the free-floating method using antibodies and avidin-biotin-peroxidase as previously described (Gaszner et al., 2004, 2009; Kita et al., 2006; Yanagita et al., 2007; Kubota et al., 2012; Otsuka et al., 2016). Briefly, after blocking endogenous peroxidase in PBS with 0.3% H_2_O_2_ and non-specific binding in 10% normal horse serum, the brain sections were incubated with primary rabbit polyclonal anti-Fos antiserum (sc-52, Santa Cruz Biotechnology, Santa Cruz, CA, USA, 1:600) diluted in 0.1 M PBS with 0.1% Triton X-100 (PBS-TX) for 16 h at room temperature. After rinsing three times for 5 min in PBS-TX, the sections were further incubated with biotinylated secondary donkey anti-rabbit IgG (AP182B, Chemicon, Temecula, CA, USA, 1:800) for 90 min at room temperature, then rinsed three times for 5 min in PBS-TX again, and finally amplified with the avidin-biotin-peroxidase complex (Vectastain ABC peroxidase kit, Vector Laboratories, Burlingame, CA, USA, 1:400) for 90 min. To detect peroxidase activity, the sections were reacted in a solution of nickel ammonium sulfate, 0.02% 3,3′-diaminobenzidene (DAB) in 0.1 M Tris-HCl buffer (pH 7.6), and 0.01% H_2_O_2_ for 20 min. Immunoreactivity for c-Fos was localized to cell nuclei and appeared as a dark gray-black stain. Sections were then washed in 0.1 M PBS, mounted, air-dried, dehydrated in graded alcohol, cleared in xylene, and coverslipped with Permount mounting medium (Fisher Scientific, Pittsburgh, PA, USA). No staining was observed on sections incubated without the primary antibody (sc-52, Santa Cruz Biotechnology, Santa Cruz, CA, USA).

To detect DCX positive cells in the hippocampus, an immunostaining was carried out according to previous studies (Lemaire et al., 2012; Pawluski et al., 2015; Requejo et al., 2015). For DCX immunostaining in the hippocampus, the adjacent series of sections was incubated with goat polyclonal anti-DCX antibody (sc-8066, Santa Cruz Biotechnology, Santa Cruz, CA, USA, 1:500) in 0.1 M PBS with 0.5% Triton X-100 and 0.5% bovine serum albumin for 24 h at room temperature. The sections were further incubated in biotinylated secondary donkey anti-goat IgG (AP180B, Chemicon, 1:1000) for 2 h at room temperature. The sections were then treated with the avidin-biotin-peroxidase complex (Vectastain ABC peroxidase kit, Vector Laboratories, 1:400) for 90 min. Avidin-biotin-peroxidase complexes were visualized using 0.02% DAB in 0.1 M Tris-HCl buffer (pH 7.6) containing 0.01% H_2_O_2_. DCX immunoreactivity was localized to the cell cytoplasm and was visible as dark-brown staining. Sections were mounted on gelatin-coated slides, air-dried, counterstained with Nissl staining for cell nuclei, dehydrated in graded alcohol, cleared in xylene, and coverslipped with Permount mounting medium. No staining was observed on sections incubated without the primary antibody (sc-8066, Santa Cruz Biotechnology, Santa Cruz, CA, USA).

### Cell Counts and Quantification

Immunoreactive cells on sections were observed using an optical microscope (BX-51, Olympus, Tokyo, Japan) equipped with a CCD camera (DP-73, Olympus). For unbiased quantification, slides were coded prior to analysis. Quantitative analysis of c-Fos immunoreactivity was performed on sections containing the DRN and PVN. Representative coronal sections of identical anatomical planes in the PVN and DRN area were selected. The total number of c-Fos positive nuclei in the entire DRN, including dorsal, ventral, and lateral regions was manually counted on sections between −7.3 and −8.3 from Bregma (corresponding to Plates 47–51 in Paxinos and Watson rat brain atlas, 4th edition). Similarly, the total number of c-Fos positive nuclei in the PVN was counted on sections between −0.8 and −2.1 from Bregma (corresponding to Plates 21–26 in Paxinos and Watson rat brain atlas). The numbers of c-Fos positive nuclei per section were calculated.

Quantitative analysis of DCX immunoreactivity was performed on sections containing the dentate gyrus (DG) in the dorsal (2–3 sections) and ventral (2 sections) hippocampus (close to −3.3 and −6.4 mm from Bregma, respectively). The caudal sections contained both the dorsal and ventral regions of the hippocampus, but the ventral region was targeted. The numbers of DCX positive cells in the granule cell layer of the DG in each region of the hippocampus were manually counted, and the densities of DCX positive cells were obtained by dividing the number of DCX positive cells by the area of the granule cell layer of the DG (mm^2^). The densities of DCX positive cells per section were calculated.

### Statistical Analysis

Data are shown as the mean ± SEM. Statistical evaluations of the experiments were investigated by applying ANOVA. Scheffe’s *post hoc* analysis was used for further analysis. Values of *p* < 0.05 were considered statistically significant. Prior to ANOVA, the normal distribution of data (Shapiro-Wilk test) and homogeneity of variance (Levene’s test) were assessed. As only data of c-Fos expression in the PVN did not show the homogeneity of variance, square root mathematical transformation was applied.

## Results

### Neuronal Activity during Acute Treadmill Running in Pre- and Post-Training

**Figure 2:**
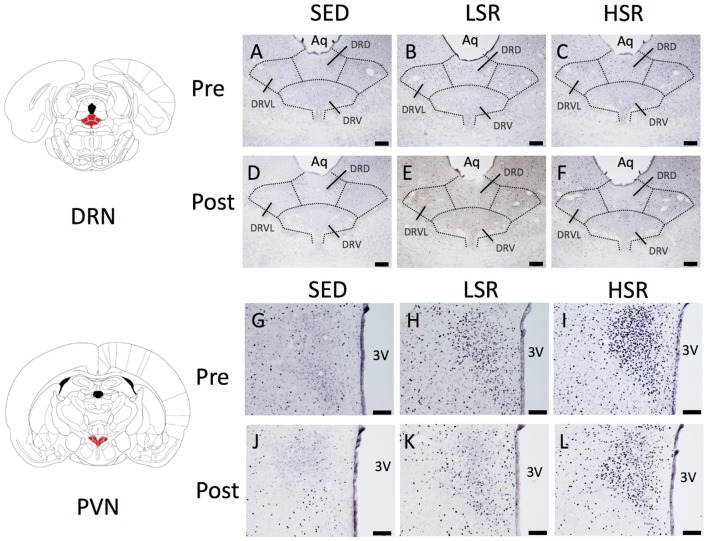
Photographs of sections immunostained for c-Fos in the dorsal raphe nucleus (DRN; **A–F**) and paraventricular nucleus (PVN; **G–L**) of rats after acute treadmill running at different intensities in the pre- and post-training groups. Scale bars are 200 μm **(A–F)** and 100 μm **(G–L)**. Aq, cerebral aqueduct; DRD, dorsal raphe, dorsal part; DRV, dorsal raphe, ventral part; DRVL, dorsal raphe, ventrolateral part; 3V, 3rd ventricle.

**Figure 3:**
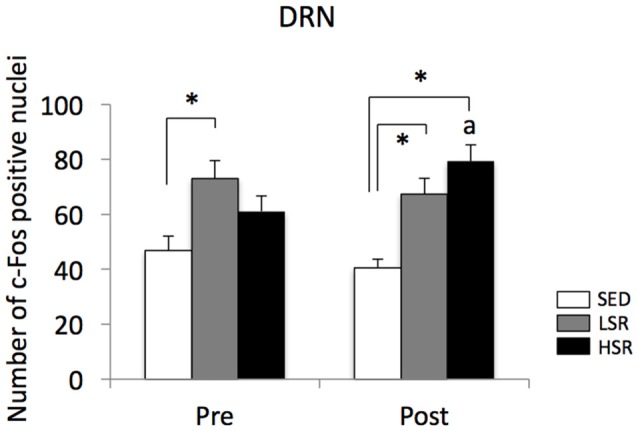
Mean (±SEM) numbers of c-Fos positive nuclei in the DRN of rats after acute treadmill running at different intensities in the pre- and post-training groups. SED, sedentary controls; LSR, low-speed runners; HSR, high-speed runners. *p < 0.05 vs. SED, ^a^*p* < 0.05 vs. HSR in the pre-training group.

We performed c-Fos immunostaining after a session of 30-min treadmill running at different speeds in the pre- and post-training groups. Representative images of c-Fos immunostaining are shown in Figure 2. In the sections of DRN, c-Fos immunoreactivity in rats that performed acute treadmill running was qualitatively higher in the ventrolateral region of the DRN (DRVL) compared to the dorsal or ventral regions of the DRN (The sub-region of the DRN is marked with the dotted line in Figure 2). The ventrolateral region of the DRN, which contains a distinct cluster of serotonergic neurons, are believed to play anti-aversive roles in stress-related functional properties (Lowry et al., 2000; Johnson et al., 2004). In the pre-training group, acute treadmill running at low speed increased c-Fos expression in the DRN, whereas high-speed treadmill running only slightly increased c-Fos expression in the DRN. In the post-training group, acute treadmill running at both low- and high-speeds increased c-Fos expression in the DRN. Two-way ANOVA (exercise intensity × training) revealed a significant main effect of exercise intensity on the number of c-Fos positive nuclei in the DRN (*F*_(2,54)_ = 15.466, *p* < 0.01) and a significant interaction (*F*_(2,54)_ = 3.218, *p* < 0.05). No significant main effect was observed for training. The simple main effect test indicated significant main effects for exercise intensity in both the pre- and post-training groups (*F*_(2,27)_ = 5.100, *p* < 0.05; *F*_(2,27)_ = 15.775, *p* < 0.01, respectively). *Post hoc* analysis showed that c-Fos expression in the pre-training group was significantly enhanced in the LSR rats, but not the HSR rats, compared to the SED rats. In the post-training group, significant increases in the number of c-Fos positive nuclei were observed in both the LSR and HSR rats compared to the SED rats. In addition, the number of c-Fos positive nuclei in the HSR rats in the post-training group was significantly larger than that in the pre-training group (Figure 3).

**Figure 4:**
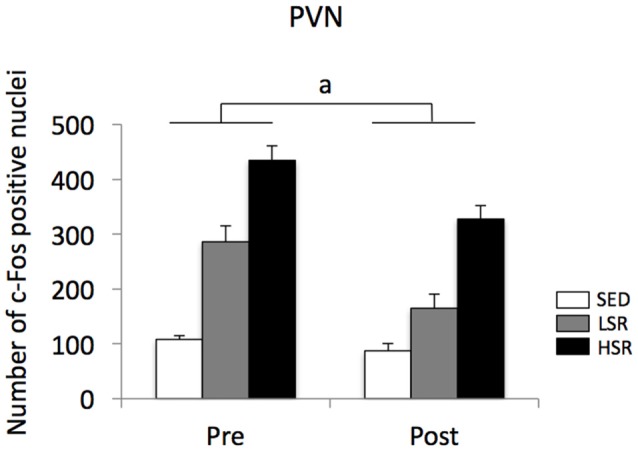
Mean (±SEM) numbers of c-Fos positive nuclei in the PVN of rats after acute treadmill running at different intensities in the pre- and post-training. SED, sedentary controls; LSR, low-speed runners; HSR, high-speed runners. ^a^*p* < 0.05 vs. the pre-training group.

In the PVN, acute treadmill running increased c-Fos expression in an exercise intensity-dependent manner in both the pre- and post-training groups, but c-Fos expression in the post-training group was generally reduced compared to that in the pre-training group (Figure 4). Two-way ANOVA (exercise intensity × training) revealed significant main effects for exercise intensity (*F*_(2,54)_ = 89.937, *p* < 0.01) and training (*F*_(1,54)_ = 21.280, *p* < 0.01) on the number of c-Fos positive nuclei in the PVN. No significant interaction was observed. *Post hoc* analysis showed that significant increases in the number of c-Fos positive nuclei were observed in both the LSR and HSR rats compared to the SED rats, and the c-Fos expression in the HSR rats was significantly higher than that in the LSR rats.

### Effects of Regular Treadmill Running on Hippocampal Neurogenesis

**Figure 5:**
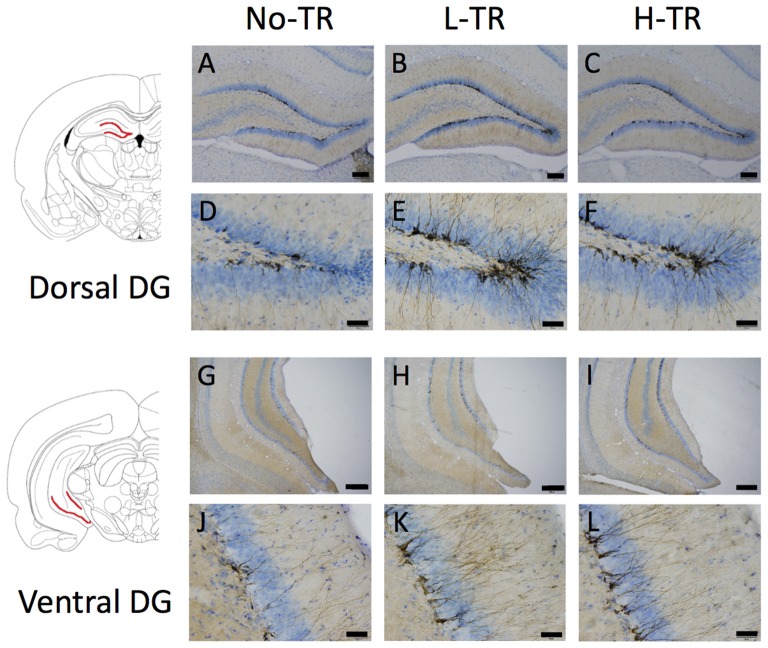
Photographs of sections immunostained for doublecortin (DCX) in the dorsal **(A–F)** and ventral **(G–L)** hippocampal dentate gyrus (DG) of rats that performed regular treadmill running at different intensities (No-TR, sedentary; L-TR, low intensity; H-TR, high intensity). High-power photomicrograph of the dorsal DG **(D–F)** and the ventral DG **(J–L)**. Scale bars are 200 μm **(A–C,G–I)** and 50 μm **(D–F,J–L)**.

**Figure 6:**
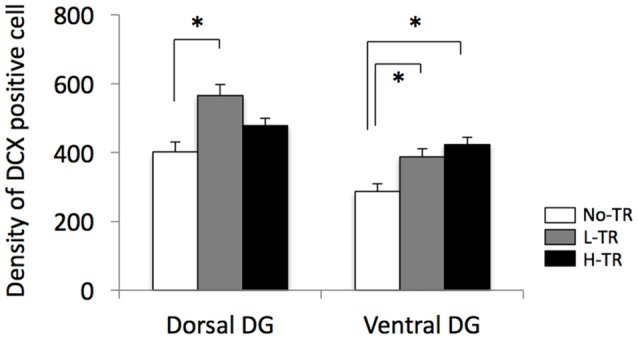
Effects of 4 weeks of regular treadmill running at different intensities on expression of DCX positive cells in the dorsal and ventral hippocampus. *p < 0.05 vs. SED.

Representative images of DCX immunostaining are shown in Figure 5. In both the dorsal and ventral hippocampal DG, DCX immunoreactivity in rats that performed regular treadmill running was qualitatively higher compared to No-TR rats, regardless of the training intensity. DCX immunoreactivity in the ventral hippocampal DG was weaker than in the dorsal hippocampal DG in both sedentary and trained rats. Two-way ANOVA (training intensity × region) revealed significant main effects for training intensity (*F*_(2,54)_ = 16.603, *p* < 0.01) and region (*F*_(1,54)_ = 34.973, *p* < 0.01) on the density of DCX positive cells (Figure 6). In addition, a significant interaction was observed (*F*_(2,54)_ = 3.288, *p* < 0.05). The simple main effect test indicated significant main effects for training intensity in both the dorsal and ventral regions (*F*_(2,27)_ = 10.264, *p* < 0.01; *F*_(2,27)_ = 9.544, *p* < 0.01, respectively). *Post hoc* analysis showed that the density of DCX positive cells in the dorsal hippocampus was significantly higher in L-TR rats compared to No-TR rats. We found no significant difference between No-TR and H-TR rats in the density of DCX positive cells (*p* = 0.07). In the ventral hippocampus, significant increases in the density of DCX positive cells were observed in both L-TR and H-TR rats compared to No-TR rats.

### Effects of Regular Treadmill Running on Performance in the Behavioral Test

**Figure 7:**
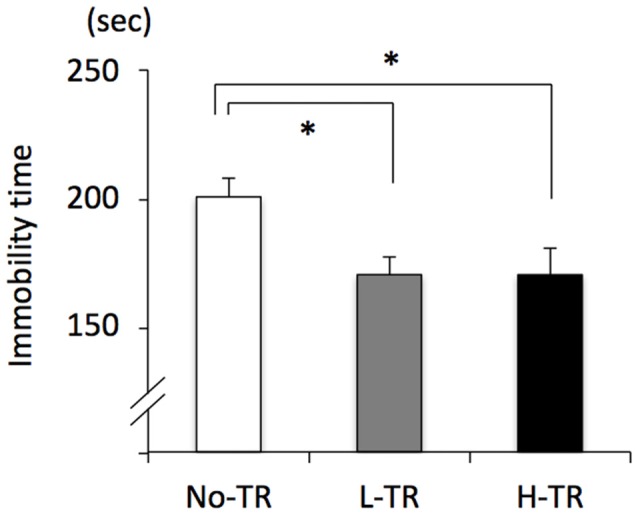
Effects of 4 weeks of regular treadmill running at different intensities on immobility time in the forced swim test. No-TR, sedentary; L-TR, low intensity; H-TR, high intensity. *p < 0.05 vs. No-TR.

Rats in the post-training group were tested for depressive-like behavior using immobility used as a measure of behavioral despair in the forced swim test. The immobility time was decreased in both L-TR and H-TR rats compared to No-TR rats (Figure 7). ANOVA revealed a significant main effect for training intensity on immobility time (*F*_(2,27)_ = 4.483, *p* < 0.05). *Post hoc* analysis revealed that the immobility time for L-TR and H-TR rats was significantly shorter than that for No-TR rats. We found no significant difference between L-TR and H-TR rats in the immobility time.

## Discussion

The present study revealed that 4 weeks of regular treadmill running modulated the sensitivity of neuronal activity in the DRN and PVN to acute exercise, and also enhanced neurogenesis in both the ventral and dorsal regions of the hippocampus regardless of the training intensity. Furthermore, the behavioral test indicated that 4 weeks of regular treadmill running resulted in decreased depressive-like behavior, regardless of the training intensity. Although these results are purely correlative and therefore no causality in these responses can be established, the results of this study suggest that enhancing the sensitivity of DRN neurons, reducing the sensitivity of neuronal activity in the PVN to acute exercise, and enhancing hippocampal neurogenesis can contribute to the antidepressant effects of regular physical exercise.

Our results showed that a session of 30-min treadmill running at low intensity (15 m/min) in both the pre- and post-training groups significantly increased neuronal activity in the DRN compared with the sedentary controls. Several studies have reported that acute physical exercise activates the central serotonergic system originating in the DRN (Chaouloff et al., 1985; Meeusen et al., 1996; Wilson and Marsden, 1996; Gomez-Merino et al., 2001). Meeusen et al. (1996) found that acute moderate treadmill running (12 m/min, 60 min) induced a significant increase in hippocampal 5-HT levels 20 min after the onset of running in rats deprived of food for 24 h, and this effect could still be observed during the first 20 min after the end of the exercise. Recently, we also found that acute low-intensity treadmill running (15 m/min) significantly enhances c-Fos expression in 5-HT neurons in the DRN (Otsuka et al., 2016). These data are consistent with the results of this study. Although our results did not determine cell types (i.e., 5-HT neuron) involved in the neural activation, our previous study has demonstrated that the c-Fos expression in 5-HT neurons in the DRN during acute exercise was tightly correlated with expression of only c-Fos positive nuclei in the DRN (Otsuka et al., 2016). Thus, it is suggested that mild exercise (i.e., lower-speed running) efficiently induce an increase in activation of 5-HT neurons in the DRN.

Although the mechanisms underlying activation of the serotonergic system by mild exercise are not precisely known, exercise-mediated lipolysis may play a key role. An increase in free fatty acids by lipolysis is accelerated by mild to moderate exercise and may induce an increase in plasma-free tryptophan, which is a precursor of 5-HT, leading to an increase in brain tryptophan and in turn, 5-HT synthesis/metabolism (Chaouloff et al., 1985; Martin, 1996). Meeusen et al. (1996) reported that acute intraperitoneal administration of tryptophan increased extracellular 5-HT and 5-Hydroxyindoleacetic acid (5-HIAA) levels in the hippocampus in rats deprived of food for 24 h and also increased extracellular 5-HT and 5-HIAA levels during mild exercise session (12 m/min) compared with saline administration. This suggests that an increase in plasma-free tryptophan during mild exercise may enhance 5-HT neuronal activity. Thus, mild exercise may be implicated in the activation of 5-HT neurons in the DRN via accelerated lipolysis.

Interestingly, after 4 weeks of regular treadmill running at high intensity (H-TR), acute high-speed running (HSR) significantly enhanced c-Fos expression in the DRN compared to the sedentary control, although enhanced c-Fos expression after acute high-speed running was not obtained in the pre-training group. This increase in neuronal activity in the DRN during acute high-speed running may be due to adaptive changes in 5-HT receptor function produced by regular treadmill running. Some studies have suggested that long-term exercise modulates 5-HT neuronal activity in the DRN by altering levels of 5-HT receptors in the DRN (Dey, 1994; Greenwood et al., 2005; Kim et al., 2015). Greenwood et al. (2005) have shown that 6-week wheel running reduces 5-HT_1B_ mRNA in the ventral DRN and increases 5-HT_1A_ mRNA in the DRN. Dey (1994) has reported that 4 weeks of swimming exercise in rats enhances sensitivity of 5-HT_2_ receptors along with a decrease in the sensitivity of 5-HT_1A_ autoreceptors. Thus, the 4 weeks of regular treadmill running at high intensity used in this study also may alter the level or sensitivity of 5-HT receptors in the DRN. This possibility should be investigated further by measuring the levels of 5-HT receptors such as 5-HT_1A_ and 5-HT_1B_ autoreceptors.

We also examined the effects of regular treadmill running at different intensities on activation of PVN neurons during acute treadmill running. Our results showed that the number of c-Fos positive nuclei in the PVN during acute treadmill running was increased in a running speed-dependent manner in both the pre- and post-training groups, and c-Fos expression in the post-training group was generally lower than that in the pre-training group. In addition, we noticed that there is a considerable c-Fos expression even in the SED groups. The SED group was placed on a stationary treadmill for 30 min with exposure to the noise of another moving treadmill without the animals. Thus, the exposure to the noise may somewhat enhance c-Fos expression in the SED groups. Several studies have indicated that acute treadmill running induces activation of PVN neurons and the HPA axis in relation to the intensity of exercise (Farrell et al., 1983; Timofeeva et al., 2003; Saito and Soya, 2004; Soya et al., 2007; Otsuka et al., 2016), suggesting that treadmill running at high intensity is a strong stressor, and low-speed running is a milder stressor. Nevertheless, 4 weeks of regular treadmill running attenuated activation of PVN neurons to acute treadmill running, regardless of the training intensity. Previous studies have suggested that long-term exercise can lead to adaptations of activation of PVN neurons, as well as the HPA axis (Dishman et al., 1998; Kawashima et al., 2004; Fediuc et al., 2006). Kawashima et al. (2004) showed that 4 weeks of treadmill running (approximately 25 m/min, 60 min/day, 5 days/week) alters CRF mRNA levels in the PVN and reduces adrenocorticotropic hormone during acute running (25 m/min, 60 min). This suggests that repeated treadmill running, even if the exercise intensity is relatively high, can correct abnormalities in the activation of CRF neurons and the HPA axis. In addition, Fediuc et al. (2006) reported that 5 weeks of voluntary wheel running progressively decreased the increased levels in circadian plasma corticosterone observed after week 1, and reaches similar values as sedentary animals by week 4. It is well known that activity of the HPA axis, including c-Fos expression in PVN neurons, habituates with repeated exposure to the same stressor (homotypic stressor; Grissom et al., 2007; Spencer and Deak, 2017). Thus, the decline in the c-Fos expression in the PVN shown in the present study may simply reflect habituation to the repeated stressor (i.e., repeated treadmill running). Another explanation for the attenuated activation of PVN neurons to acute treadmill running in the post-training group may be a relative reduction in the intensity of the acute running. The training protocol used in this study may improve the aerobic capacity (i.e., increase the LT), and thereby, the rats in the post-training group may perform the acute treadmill running at relatively lower intensity compared to the pre-training group, and this may attenuate neuronal activity in the PVN. Further studies will be needed to clarify the mechanisms by which regular treadmill running attenuates the sensitivity of PVN neurons.

Furthermore, we investigated the effects of regular treadmill running at different intensities on hippocampal neurogenesis and found that regular treadmill running enhanced DCX immunoreactivity in the ventral region of the hippocampus compared with No-TR, regardless of the training intensity. DCX immunoreactivity in the dorsal region of the hippocampus was significantly higher in L-TR, but not H-TR rats (*p* = 0.07), compared to No-TR rats. Our knowledge about the pathophysiology of depression is not complete, but abnormal hippocampal neurogenesis, as well as dysregulation of the HPA axis and deficiency in central monoamine transmission has been implicated in symptoms of depression (Jacobs, 2002; Berton and Nestler, 2006; Pittenger and Duman, 2008; Balu and Lucki, 2009). Several neuroimaging studies of depressed patients have found a reduction in the volume of the prefrontal cortex and hippocampus, which are thought to mediate the cognitive aspects of depression (Drevets, 2001; Harrison, 2002). Likewise, hippocampal neurogenesis may play a role in antidepressant effects (Dranovsky and Hen, 2006; Becker and Wojtowicz, 2007; Snyder et al., 2011). Using transgenic or radiation methods to inhibit adult neurogenesis, Snyder et al. (2011) have suggested that adult hippocampal neurogenesis may help to buffer stress responses and depressive behavior. In addition, extensive studies have shown that physical exercise has beneficial effects on mood, stress responses, and stress-related psychiatric disorders such as depression (Paluska and Schwenk, 2000; Duman et al., 2008; Greenwood and Fleshner, 2011) and has potent impacts on promoting the function of the hippocampus and stimulating hippocampal neurogenesis (van Praag et al., 1999; Lou et al., 2008; Choi et al., 2011; Garrett et al., 2012; Okamoto et al., 2012; Kim and Seo, 2013). Therefore, exercise-induced hippocampal neurogenesis may be an important factor in the antidepressant effects of exercise.

Many previous studies have reported that voluntary exercise using wheel running increases hippocampal neurogenesis, but only a few studies have reported the effects of forced running (i.e., treadmill running) on hippocampal neurogenesis. Okamoto et al. (2012) examined the effect of treadmill running at below-LT (13.5 m/min) or supra-LT (28 m/min) on adult hippocampal neurogenesis in rats, and showed that after 2 weeks of the treadmill running (30 min/day), hippocampal neurogenesis was enhanced with below-LT exercise, but not with supra-LT exercise. Lou et al. (2008) also reported that 1 week of low- or moderate-intensity exercise (11 m/min), but not high-intensity exercise (22 m/min), on a treadmill running task (30 min/day) enhances neurogenesis in the DG of the hippocampus. Taken together with our results, mild treadmill running may efficiently enhance hippocampal neurogenesis. Nevertheless, our results showed that even regular treadmill running at high intensity significantly increased neurogenesis in the ventral region of the hippocampus. This discrepancy may be partially due to the differences in training regimens, because the training regimens used in the previous studies consisted of relatively shorter periods of training or running. Thus, long-term exercise, even if the exercise is regular forced running, may enhance hippocampal neurogenesis, regardless of the training intensity.

Adult hippocampal neurogenesis is implicated in monoaminergic transmission including transmission by serotonergic neurons (Malberg et al., 2000; Jacobs, 2002; Santarelli et al., 2003; Balu and Lucki, 2009). Some studies have reported that chronic treatment with antidepressants including SSRIs modulates depressive-like behavior accompanied by a selective increase in neurogenesis in the ventral region of the hippocampus, which critically regulates mood (Banasr et al., 2006; Boldrini et al., 2009; Fanselow and Dong, 2010; Kheirbek and Hen, 2011). Klempin et al. (2013), using tryptophan hydroxylase 2-deficient mice that lack brain 5-HT, showed that the release of central 5-HT is critical for exercise-induced adult hippocampal neurogenesis. In the present study, we found that in the post-training group, a session of treadmill running at not only low but also high speed could activate DRN neurons. Thus, it may be indicated that regular exercise would enhance the sensitivity of 5-HT neurons in the DRN to acute exercise, and thereby increase neurogenesis. In this study, however, we noticed that the c-Fos expression after the acute exercise was seen predominantly in the DRVL. Although recent functional neuroanatomical studies have suggested that 5-HT neurons located in the ventrolateral and intrafascicular regions of the DRN (DRVL, DRI) are implicated in anti-panic and/or antidepressant responses (Hale et al., 2012; Nicastro and Greenwood, 2016), it has been reported that the DRVL, but not DRI, does not project to the hippocampus (Muzerelle et al., 2016). These suggest that the increase in hippocampal neurogenesis produced by regular exercise may be due to activation of serotonergic neurons located in regions other than DRVL or to non-serotonergic mechanisms. Further investigations are needed to clarify the site specificity, training regimen dependency, or role of serotonergic system for hippocampal neurogenesis enhanced by regular exercise.

On the other hand, it has been indicated that cell proliferation and neurogenesis in the hippocampus is inhibited by prolonged stress and excess glucocorticoids, which implicate activation of the HPA axis (Mirescu and Gould, 2006; McEwen, 2007). In the present study, we found that in the post-training group, a session of treadmill running at both low and high speeds induced attenuated activation of PVN neurons compared with the pre-training group. Thus, the 4 weeks of regular treadmill running used in this study may mediate hippocampal neurogenesis through changes in the sensitivity of CRF neurons in the PVN, as well as the HPA axis, to acute exercise.

Lastly, the present study indicated that immobility time in the forced swim test was decreased after 4 weeks of forced running (i.e., treadmill running) compared to No-TR, regardless of the training intensity. The treadmill training may reduce immobility time in the forced swim test by increasing strength or locomotor activity. We also recorded the immobility time during the first 6 min (excluding the first 1 min) in the initial 15-min swim session which was conducted on day 1, and consequently no significant difference among the training intensity (including No-TR) was observed in the immobility time (data not shown). This suggests that an increase of strength by the treadmill training scarcely affected the immobility time in the rats of the present study. In the laboratory animal model of regular exercise for examining the beneficial effects of physical exercise on stress-related psychiatric disorders including depression, many studies have utilized free access to voluntary exercise such as wheel running (Duman et al., 2008; Greenwood and Fleshner, 2011), and several studies suggest that voluntary exercise may be more beneficial for stress-related psychiatric disorders. Interestingly, our results showed that even regular forced running (i.e., treadmill running) resulted in decreased depressive-like behavior, regardless of the training intensity. Treadmill training restricts exercise controllability of the animals, thereby may lead to potential confounding effects of forced exercise such as adaptation to a chronic stressor. Our results indicated that the treadmill training could produce enhancement of hippocampal neurogenesis and attenuation in the sensitivity of neuronal activity in the PVN to acute exercise, as well as decrease in depressive-like behavior, suggesting that the treadmill training used in this study may result in the potentially positively adaptations. In addition, the use of treadmill running allows us to easily define treadmill training in terms of a specific type, duration and intensity of exercise. One goal of this study was to understand which features of exercise contribute to the antidepressant effects of exercise, and we focused on the intensity in treadmill training as one potential factor. However, our data suggest that repeated exposure to the same exercise seems to be more important factor rather than the training intensity. Some previous studies reported that forced exercise such as treadmill training did not reduce anxiety- or depressive-like behaviors in rodents (Burghardt et al., 2004; Leasure and Jones, 2008; Greenwood et al., 2013). This discrepancy may be due to some factors, including differences in the training protocol, period of the light/dark cycle, and sex of the animals. In the previous Leasure and Jones (2008) and Greenwood et al. (2013) studies, the intensity or duration of daily forced exercise during the training period was not constant (5–50 min, 10–20 m/min; programmed running cycle, 5–30 m/min, respectively). The uncontinuous running protocol may be stressful due to a change in expected routines (Skalicky and Viidik, 1999), thereby may be less effective in the behavioral changes. Burghardt et al. (2004) used 4 and 8 weeks of forced treadmill running for 45 min/day, 5 days/week at 20 m/min as the training protocol, and running took place during the early light period of the light/dark cycle. Some studies have suggested that hormonal responses, including cortisol levels can be influenced by the time of exercise training (i.e., time-of-day effect; Seo et al., 2013). Thus, the time-of-day effect may influence the behavioral changes by exercise. Further investigation of the features of exercise producing the antidepressant effects is needed to establish the optimal regimens of physical exercise.

In conclusion, our results showed that regular treadmill exercise modulates the activity of DRN and PVN neurons during acute treadmill running. In addition, regular exercise enhanced hippocampal neurogenesis and decreased depressive-like behavior, regardless of the training intensity. Although this study is purely correlative and therefore no causality can be established, these results suggest that long-term repeated exercise, regardless of the training intensity, may improve depressive-like behavior by modulating neuronal activity in the DRN and PVN during acute exercise, as well as enhancing hippocampal neurogenesis.

## Author Contributions

AN, SA, NK, TN and IK designed research; analyzed data. AN and IK performed research; wrote the article.

## Conflict of Interest Statement

The authors declare that the research was conducted in the absence of any commercial or financial relationships that could be construed as a potential conflict of interest.
